# Leaflet thickening and stent geometry in sutureless bioprosthetic aortic valves

**DOI:** 10.1007/s00380-020-01553-9

**Published:** 2020-01-16

**Authors:** Raquel Themudo, Mikael Kastengren, Elin Bacsovics Brolin, Kerstin Cederlund, Anders Svensson, Magnus Dalén

**Affiliations:** 1 Department of Radiology, Karolinska University Hospital, Stockholm, Sweden; 2 Department of Clinical Physiology, Karolinska Institutet, Stockholm, Sweden; 3 Department of Cardiology, Karolinska University Hospital, C11:28, 171 76 Stockholm, Sweden; 4 Department of Molecular Medicine and Surgery, Karolinska Institutet, Stockholm, Sweden; 5 Department of Clinical Science, Intervention and Technology, Karolinska Institutet, Stockholm, Sweden; 6 Department of Radiology, Capio S:t Göran Hospital, Stockholm, Sweden; 7 Department of Cardiac Surgery, Karolinska University Hospital, Stockholm, Sweden

**Keywords:** Aortic valve surgery, Bioprosthesis, Four-dimensional cardiac computed tomography, Leaflet thickening, Stent geometry

## Abstract

Underexpansion of transcatheter heart valves and the surgically implanted Perceval sutureless aortic valve bioprosthesis has been suggested as an underlying mechanism for hypo-attenuated leaflet thickening (HALT). This was a single-center prospective observational study that included 47 patients who underwent surgical aortic valve replacement with the Perceval sutureless bioprosthesis (LivaNova, London, United Kingdom) from 2012 to 2016 and were studied by four-dimensional cardiac computed tomography (CT). The association between overall and regional expansion and the prevalence of HALT was analyzed. In total 46 patients were included in the analysis. HALT was found in 39.1% of patients and the mean overall prosthesis expansion was 75.5 ± 5.2% (range 64.6–84.8%). Overall expansion did not differ between patients with HALT compared with patients without HALT (mean overall expansion 74.0 ± 5.2% vs. 76.5 ± 5.0%, *P* = 0.11). The prevalence of HALT was lower in patients with overall expansion > 80% compared to patients with expansion < 80% expansion though not significantly (20% vs. 44.4%, *P* = 0.16). None or trivial regional underexpansion was found in 94.7% of coronary cusps. There was no significant association between regional underexpansion and the prevalence of HALT (mean coronary cusp angle 120 ± 8° vs. 119 ± 10°, *P* = 0.53). The prevalence of HALT and overall underexpansion was high in the Perceval sutureless bioprosthetic valve. Overall underexpansion was not associated with HALT. Whether severe overall underexpansion increases the risk for HALT requires further study. Regional underexpansion was uncommon in the Perceval sutureless bioprosthetic valve and not associated with HALT.

*Clinical trial registration* Unique identifier: NCT03753126 (http://www.clinicaltrials.gov).

## Introduction

Hypo-attenuated leaflet thickening (HALT), with or without associated reduced leaflet motion, is prevalent in both transcatheter and surgically implanted bioprosthetic aortic valves [1–3]. The underlying cause has been speculated to be thrombogenic as anticoagulation therapy with warfarin has been associated with restored cusp thickness in patients with HALT [2]. The prevalence of HALT has been associated with cardiovascular events [2, 4].

Recently, bioprosthetic valve underexpansion has been hypothesized to affect leaflet thickening in aortic bioprosthetic valves [5]. In transcatheter valves, regional underexpansion of transcatheter aortic valve cusps has been reported to be associated with an increased prevalence of HALT [5]. In the surgically implanted Perceval sutureless aortic valve bioprosthesis, overall underexpansion of the valve and failure to achieve circularity has been speculated to cause HALT [6, 7].

The aim of this study was to investigate the potential relationship between four-dimensional (4D) cardiac computed tomography (CT)-derived overall and regional underexpansion and HALT in the Perceval sutureless bioprosthetic valve.

## Methods

### Study design

This was a single-center prospective observational study approved by the regional Human Research Ethics Committee, Stockholm, Sweden. Informed consent was obtained from patients meeting the inclusion criteria.

### Study group

All patients who had undergone surgical aortic valve replacement (AVR) with implantation of the Perceval sutureless bioprosthesis (LivaNova, London, United Kingdom) at Karolinska University Hospital in Stockholm, Sweden (October 2012 to February 2016) were eligible. The criterion to implant the Perceval sutureless bioprosthesis was aortic stenosis with indication for primary isolated non-emergent AVR. Implantation was considered feasible if the ratio between the diameter of the sinotubular junction and the diameter of the aortic annulus did not exceed 1.3. A type 0 bicuspid aortic valve was a contraindication for Perceval sutureless bioprosthesis implantation. Exclusion criteria were severely impaired renal function (glomerular filtration rate < 30 mL min^−1^/1.73 m^2^), unwillingness to undergo CT examination, or inability to participate in the examination for logistical reasons.

### Sutureless surgical aortic valve replacement

Patients underwent sutureless bioprosthesis AVR via either full sternotomy or partial upper hemisternotomy. Cardiopulmonary bypass was established with central arterial and central or peripheral percutaneous venous cannulation. Implantation was performed as previously described [8].

### Antithrombotic regime

According to the standard antithrombotic protocol for aortic bioprostheses at our center, postoperative antithrombotic treatment consisted of low molecular weight heparin until full mobilization and life-long treatment with acetylsalicylic acid 75 mg once daily. Patients without a preoperative indication for oral anticoagulation did not receive oral anticoagulants postoperatively. In patients preoperatively treated with long-term oral anticoagulation, this treatment was paused 3 days prior to the operation without bridging with low molecular weight heparin. In these patients, anticoagulation therapy (without additional acetylsalicylic acid) was re-administered at day 1 postoperatively.

### Cardiac computed tomography data acquisition

Oral metaprolol (50–100 mg according to heart rate) was administered 1 h prior to the CT examination. All patients were scanned using a dual-source 2 × 64 row multidetector computed tomograph (Siemens Somatom Definition Flash; Siemens Healthcare, Forchheim, Germany). Scanning was performed with retrospective ECG scanning, 100–120 kVp depending on patient’s weight, automatic dose modulation (CARE dose) and full-dose R–R scanning (no ECG mA modulation), 64 × 0.6 mm detector collimation, 0.28 s rotation time (75 ms temporal resolution). An intravenous contrast agent with a concentration of 320 mg iodine per mL (Visipaque, GE Healthcare, Stockholm, Sweden) was used with a fixed injection time of 8 s and a dose of 200 mg iodine/kg body weight. A contrast dose increase of 20% was used for patients scanned with 120 kVp. Delay time was defined using the test bolus technique. Data were reconstructed to 0.75 mm slice thickness with an increment of 0.4 mm at 5% intervals (20 phases) throughout the R–R interval and at best systolic and diastolic phases. A more detailed description of CT data acquisition has previously been described [3]. For in vitro assessment of the Perceval sutureless bioprosthesis in each size, we used the same computed tomograph and scanning parameters as for the in vivo CT examinations.

### Cardiac computed tomography analysis

**Fig. 1:**
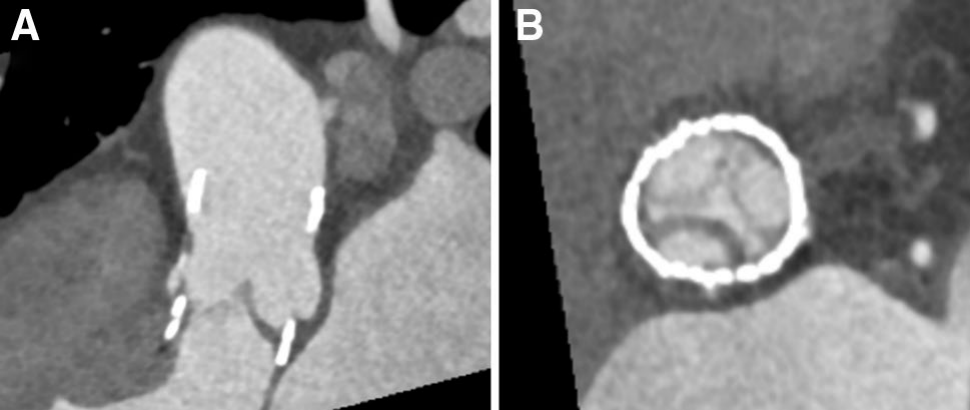
Cardiac computed tomography multiplanar reformatted reconstructions of a perceval sutureless aortic valve bioprosthesis in mid-diastole. One cusp was markedly thickened with hypo-attenuated leaflet thickening (panel A). The three valve leaflets are shown simultaneously: two of them normal and one cusp with hypo-attenuated leaflet thickening (panel B)

CT data analysis was performed using the Syngo.via software (Siemens Healthcare) on a PACS workstation. 4D cardiac CT images were analyzed independently by two experienced readers (level 2 according to American College of Cardiology (ACC) Foundation/American Heart Association (AHA) levels of CT reading competence) [9]. Joint readings involving a third experienced reader were subsequently performed to reach a consensus. For assessment of leaflet anatomy, 1-mm-thick multiplanar reformatted reconstruction (MPR) images in the plane of the leaflet and in a perpendicular plane to the leaflet being assessed were used. Still images from selected phases of the cardiac cycle, as well as dynamic images of the entire cardiac cycle, were used. An examination was considered non-diagnostic if artifacts prevented reliable assessment of one or more valve leaflet. HALT was defined according to the definition previously proposed [10]: evidence of one or more leaflet with hypo-attenuated thickening identifiable in at least two different MPR projections. HALT is illustrated in Fig. 1.

Bioprosthetic valve expansion was analyzed at the valvular level in the end-diastolic phase of the heart cycle using 1-mm-thick MPR images. Overall bioprosthetic valve geometry was measured with regard to maximal and minimal stent diameters (mm) and cross-sectional area (mm^2^). In vitro measurements were made for each available size (small, medium, large, extra large) of the Perceval sutureless bioprosthesis using CT images to assess maximal prosthesis dimensions. The degree of overall expansion of the implanted prosthesis was calculated as the ratio between the in vivo cross-sectional area (CSA) and the in vitro CSA of the implanted valve according to the formula (in vivo CSA/in vitro CSA) × 100 [5, 7]. The degree of eccentricity (%) was calculated as ([max diameter − min diameter]/max diameter) × 100 [5].

**Fig. 2:**
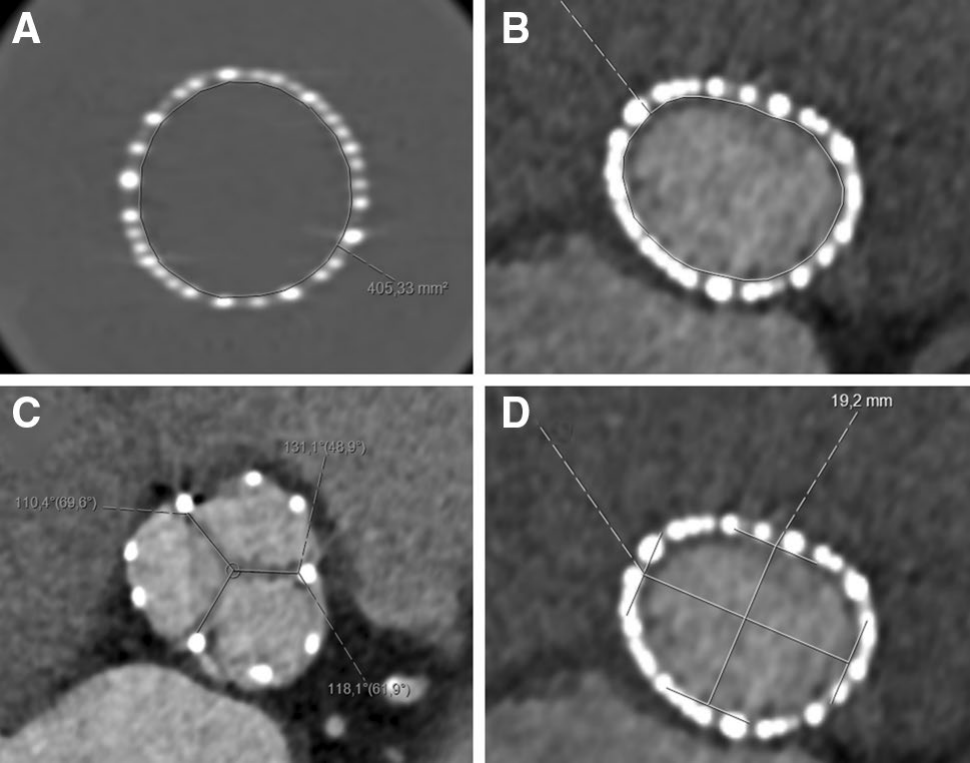
Cardiac computed tomography analyses. **a** Measurement of the in vitro cross-sectional area in a Perceval bioprosthetic valve. **b** Measurement of in vivo cross-sectional area in an implanted bioprosthesis. The degree of overall expansion of the implanted prosthesis was calculated as the ratio between the in vivo cross-sectional area (CSA) and the in vitro CSA of the implanted valve according to the formula (in vivo CSA/in vitro CSA) × 100. **c** Regional expansion measured using the angle (°) formed by the border of each prosthetic leaflet and the center point of the valve. **d** The degree of eccentricity (%) was calculated as ([max diameter–min diameter]/max diameter) × 100

Regional expansion was measured at the coaptation level in the end-diastolic phase of the heart cycle using 1-mm-thick MPR images, using the angle (°) formed by the border of each prosthetic leaflet and the center point of the valve. This angle was measured for all three prosthetic leaflets (non-coronary cusp [NCC], right coronary cusp [RCC], and left coronary cusp [LCC]). Normal regional valve expansion was defined as an expansion angle > 114° (120° with a 5% error margin). Regional valve underexpansion was defined as trivial if angle 102°–114°, mild if angle 90°–102°, moderate if angle 78°–90°, and severe if angle < 78° [5]. CT images illustrating how overall expansion, regional expansion, and eccentricity were measured and calculated are presented in Fig. 2.

### Study outcomes

Primary outcomes were overall and regional underexpansion of the bioprosthetic valve. Secondary outcome was eccentricity of the bioprosthetic valve.

### Statistical methods

Variables were described using frequencies and percentages for categorical variables, and means and standard deviations (SD) for continuous variables. Continuous variables were compared using the *t* test, and categorical or binary variables were compared using Pearson’s *χ*^2^ test. A two-sided *P* value of less than 0.05 was considered to indicate statistical significance. Statistical analyses were performed using Stata 15.1 (Stata Corp, College Station, TX, USA).

## Results

### Study group

Between October 2012 and February 2016, a total of 58 patients received a Perceval sutureless bioprosthesis. One patient died before initiation of the study at postoperative day 127. Fifty-seven patients were eligible for participation in the study. Ten patients were not included in the study (one with impaired renal function, seven unwilling to participate in the study, two who were indisposed for logistical reasons). 4D cardiac CT was performed between February and March 2016 in 47 patients. All 4D cardiac CT examinations were diagnostic regarding the evaluation of HALT. One examination was non-diagnostic for overall expansion of the bioprosthesis and this patient was excluded. Three examinations were non-diagnostic for regional bioprosthesis expansion due to motion artifacts at the end-diastolic phase of the heart cycle and these patients were excluded. Consequently, a total of 46 patients were included in the analysis of overall underexpansion, and 44 patients were included in the analysis of regional underexpansion. Patient characteristics at the time of surgery are shown in Table 1. 4D cardiac CT was performed at a median of 491 days (quartile 1: 287, quartile 3: 933 days) postoperatively.

**Table 1:** Patient characteristics and prosthesis size

	Total population (*n* = 46)	No HALT (*n* = 28)	HALT (*n* = 18)	*P* value
Age, years, mean ± SD	74.5 ± 5.5	75.7 ± 4.0	72.8 ± 7.8	0.092
Female sex	35 (76%)	21 (75%)	14 (78%)	0.83
Body mass index, kg/m^2^, mean ± SD	27.8 ± 5.0	27.7 ± 3.8	28.1 ± 6.5	0.80
Left ventricular ejection fraction ≤ 50%	43 (93%)	26 (93%)	17 (94%)	0.83
Estimated glomerular filtration rate, mL min^−1^ 1.73 m^−2^, mean ± SD	67.5 ± 18.2	68.9 ± 16.0	65.2 ± 21.5	0.51
Diabetes mellitus	10 (22%)	4 (14%)	6 (33%)	0.13
Hypertension	33 (72%)	20 (71%)	13 (72%)	0.95
Cerebrovascular event	6 (13%)	4 (14%)	2 (11%)	0.76
Chronic lung disease	4 (9%)	4 (14%)	0	0.09
Peripheral artery disease	0	0	0	–
Previous myocardial infarction	1 (2%)	0 (0%)	1 (6%)	0.21
Previous cardiac surgery	1 (2%)	1 (4%)	0	0.42
EuroSCORE II, mean ± SD	2.03 ± 1.08	2.10 ± 1.18	1.91 ± 0.92	0.57
Prosthesis size				0.41
Small	4 (9%)	4 (14%)	0	
Medium	17 (37%)	10 (36%)	7 (39%)	
Large	20 (43%)	11 (39%)	9 (50%)	
Extra large	5 (11%)	3 (11%)	2 (11%)	

Data are *n* (%) unless otherwise noted

*EuroSCORE II* European System for Cardiac Operative Risk Evaluation Score II, *HALT* hypo-attenuated leaflet thickening, *SD* standard deviation

### Hypo-attenuated leaflet thickening

HALT was found in 18 (39.1%) patients, of which 10 (55.6%) had one affected leaflet, six (33.3%) had two affected leaflets, and two (11.1%) had HALT of all three leaflets. As a result, 28/132 leaflets (21.2%) were found to have HALT (10 RCC, 8 LCC, and 10 NCC).

### Overall expansion

**Fig. 3:**
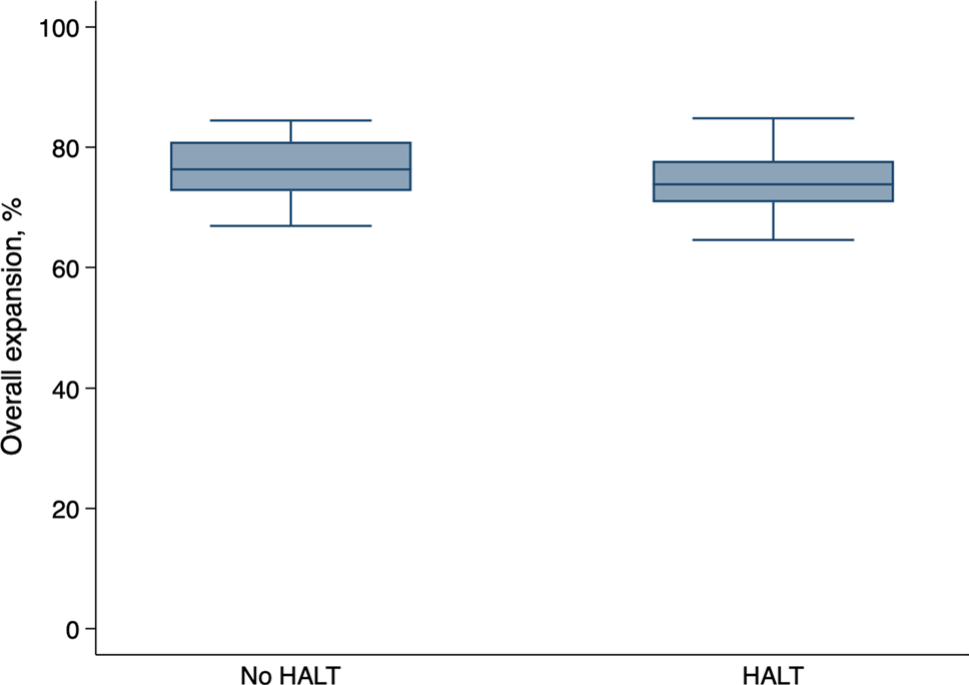
Overall expansion of the implanted prosthesis in relation to hypo-attenuated leaflet thickening. The whiskers denote the lowest/highest data points still within 1.5 interquartile range of the lower/upper quartile

**Fig. 4:**
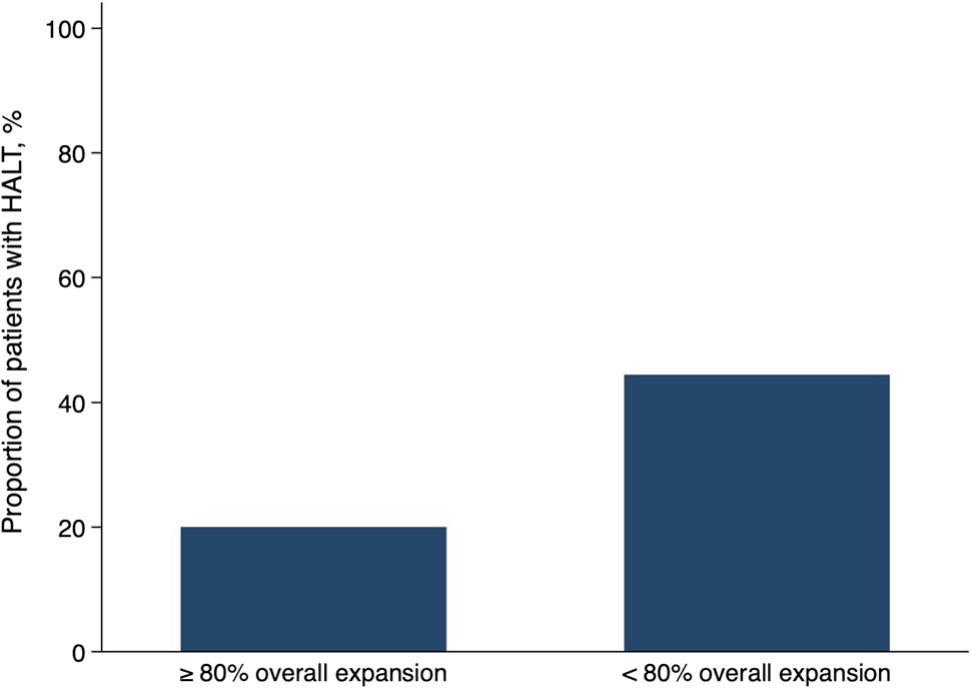
Proportion of patients with hypo-attenuated leaflet thickening in patients with < 80% overall expansion versus patients with ≥ 80% overall expansion of the implanted prosthesis

The mean overall prosthesis expansion was 75.5 ± 5.2% in the total population. There was no significant association between overall expansion and the prevalence of HALT (74.0 ± 5.2% in patients with HALT vs. 76.5 ± 5.0% in patients without HALT, *P* = 0.11; Fig. 3). As evaluated on the CT examination, ten patients (21.7%) reached an overall expansion of ≥ 80%. The prevalence of HALT was lower in patients with overall expansion > 80% compared to patients with expansion < 80% expansion though not significantly (20% vs. 44.4%, *P* = 0.16; Fig. 4).

### Regional underexpansion

**Fig. 5:**
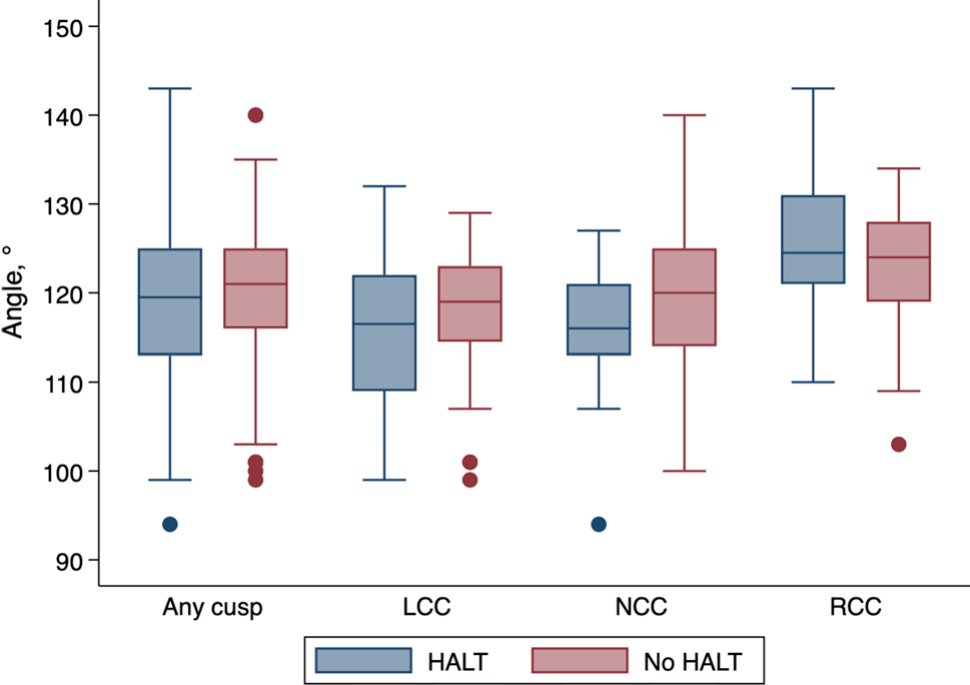
Regional expansion, measured using the angle formed by the border of each prosthetic leaflet and the center point of the valve, in relation to hypo-attenuated leaflet thickening, in each coronary cusp. The whiskers denote the lowest/highest data points still within 1.5 interquartile range of the lower/upper quartile. The points denote outliers. *LCC* left coronary cusp, *NCC* non-coronary cusp, *RCC* right coronary cusp

The prevalence of regional underexpansion is presented in Table 2. There was no significant association between regional underexpansion and the prevalence of HALT (Fig. 5, Table 2).

**Table 2:** Relation between hypo-attenuated leaflet thickening and regional underexpansion of coronary cusps

	Any cusp			RCC			LCC			NCC		
	No HALT (*n* = 104)	HALT (*n* = 28)	*P* value	No HALT (*n* = 34)	HALT (*n* = 10)	*P* value	No HALT (*n* = 36)	HALT (*n* = 8)	*P* value	No HALT (*n* = 34)	HALT (*n* = 10)	*P* value
Regional expansion, °, mean ± SD	120 ± 8	119 ± 10	0.53	123 ± 7	126 ± 9	0.35	118 ± 7	116 ± 10	0.55	120 ± 9	115 ± 9	0.13
Regional underexpansion classification			0.70			0.88			0.77			0.71
None	82 (79%)	20 (71%)		30 (88%)	9 (90%)		27 (75%)	5 (62%)		25 (74%	6 (60%)	
Trivial	17 (16%)	6 (21%)		4 (12%)	1 (10%)		6 (17%)	2 (25%)		7 (21%)	3 (30%)	
Mild	5 (5%)	2 (7%)		0	0		3 (8%)	1 (12%)		2 (6%)	1 (10%)	
> Mild	0	0		0	0		0	0		0	0	

Data are *n* (%) unless otherwise noted

*HALT* hypo-attenuated leaflet thickening, *LCC* left coronary cusp, *NCC* non-coronary cusp, *RCC* right coronary cusp, *SD* standard deviation

### Eccentricity

**Fig. 6:**
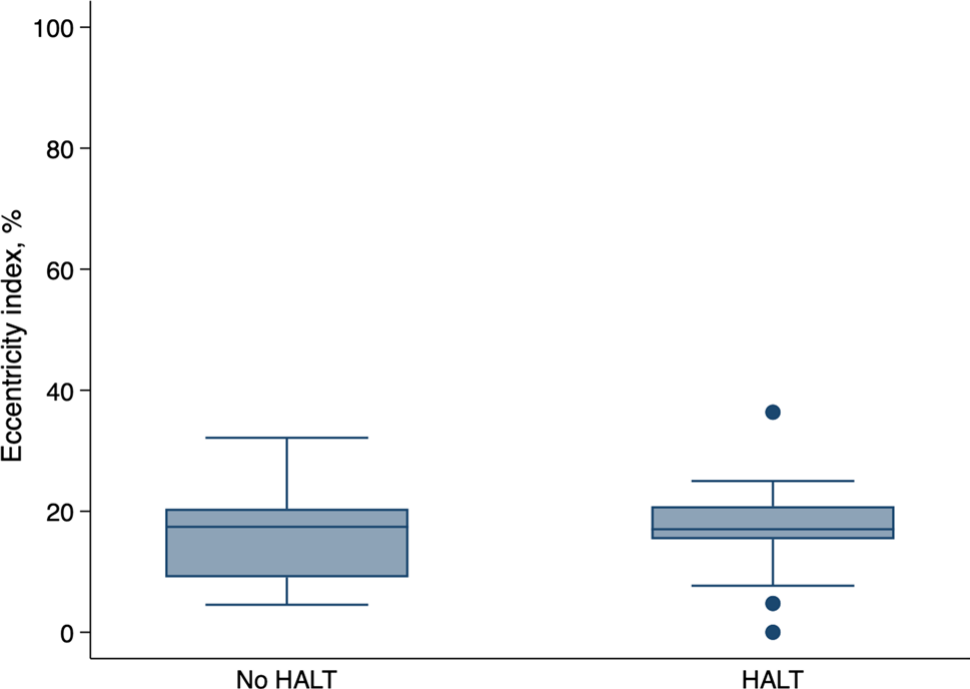
The degree of eccentricity of the implanted prosthesis in relation to hypo-attenuated leaflet thickening. The whiskers denote the lowest/highest data points still within 1.5 interquartile range of the lower/upper quartile. The points denote outliers

The mean eccentricity index was 16.4 ± 7.7% in the total population. There was no significant association between eccentricity index and the prevalence of HALT (17.0 ± 8.1% in patients with HALT vs. 16.0 ± 7.6% in patients without HALT, *P* = 0.66; Fig. 6). Eccentricity index of more than 15% was similar in patients with and without HALT (78% vs. 61%, *P* = 0.23).

## Discussion

In this study, the prevalence of HALT and overall underexpansion was high but the prevalence of regional underexpansion low in the Perceval sutureless bioprosthetic valve. Patients with an overall expansion < 80% had a higher incidence of HALT though not significantly. To determine if severe oversizing increases the risk for HALT in this prosthesis would require further study. Regional underexpansion in the Perceval sutureless bioprosthetic valve was not associated with HALT.

HALT has been found to occur in both transcatheter and surgical bioprosthetic aortic valves. Studies have shown a varying prevalence ranging from < 5% to as high as 40% [1, 11]. In a large observational study of 931 patients, HALT occurred more commonly in TAVI patients compared to patients receiving surgical aortic valves (13% vs. 4%) [2]. In our study, the prevalence of HALT was high (39.1%). An explanation could be that CT examinations in our study where performed after a median of 491 days which is relatively late compared to previous studies. Leaflet thrombosis has been speculated to be the underlying cause of HALT. The phenomenon is less prevalent in patients on anticoagulation therapy with warfarin [2] and the condition has been shown to be at least temporarily reversible when treated with warfarin or novel oral anticoagulants [12]. However, aggressive anticoagulation strategies can be associated with an increased risk of adverse events which have to be weighed against the clinical implications of HALT. In a recent trial, TAVI patients treated with Rivaroxaban had increased risks of all-cause mortality, thromboembolic events, and bleeding compared to those on antiplatelet therapy [13].

Recent findings suggest that HALT may affect clinical outcomes. A meta-analysis including 6 studies and a total 1704 patients showed that cerebrovascular events, defined as a composite of stroke or transient ischaemic attack, occurred significantly more frequently in patients with subclinical leaflet thrombosis [4]. Furthermore, it has been speculated that HALT may be related to reduced leaflet durability as prosthetic valve thrombosis is associated with prosthesis dysfunction and reduced durability [11, 14].

There is not sufficient research to explain why some valves or leaflets would be more prone to HALT than others. However, it has been suggested that underexpansion of the valve could be associated with leaflet anomalies. A study that included 75 patients who underwent TAVI with three different transcatheter valves showed an increased prevalence of HALT in patients with regional TAVI valve underexpansion, hypothesizing that an underexpanded “wrinkled” leaflet is more prone to thrombosis on its surface. Regional underexpansion was prevalent in 31–37% of the leaflet sites, depending on the type of transcatheter valve [5]. Other possible causes of HALT such as trauma to the valve before or during implantation and inflammatory response related to the procedure remain to be determined [15]. In the surgically implanted bioprostheses in the present study, regional underexpansion was rare. When present, regional underexpansion was mild and was not associated with a higher prevalence of HALT. This might be explained by the removal of the native aortic valve and decalcification of the aortic annulus performed in surgical aortic valve replacement. This procedure likely limits the risk of asymmetrical valve expansion owing to uneven areal distribution of residual aortic valve and annular calcification.

In surgically implanted Perceval sutureless bioprostheses, overall underexpansion and failure to achieve circularity have been hypothesized to affect leaflet function [6]. One study showed that the degree of oversizing of the implanted prosthesis (calculated as the ratio between the native aortic annulus CSA and the in vitro CSA of the implanted prosthesis), and thereby overall underexpansion, was the most important predictor for increased postoperative transprosthetic gradients [7]. In the present study, there was no association between overall expansion or eccentricity of the bioprosthesis and the prevalence of HALT. In the TAVI population, an oversizing > 20% is rarely accepted and is considered severe [16]. In our study, only ten patients (21.7%) reached an acceptable overall expansion of ≥ 80% of the prosthesis. HALT was less common in patients with overall expansion ≥ 80% compared to patients with < 80% overall expansion though not significantly. General oversizing of the Perceval sutureless prosthesis have been previously reported and suggestions to modify the sizing process to adjust for this [7] have been implemented.

This was a single-center observational study with the aim to investigate the potential relationship between overall and regional underexpansion and HALT in the Perceval sutureless bioprosthetic valve. It was not designed to find predictors of, nor adverse events associated with, HALT. Patients were not included in the study at the time of valve replacement and, therefore, the time intervals between AVR and 4D cardiac CT examinations varied considerably. However, very few previous studies have investigated the potential association between bioprosthetic valve geometry and HALT. The Perceval sutureless prosthesis has similarities with both conventional surgical as well as transcatheter aortic bioprostheses, making investigation of causes of HALT of this prosthesis of particular interest.

In conclusion, the prevalence of HALT and overall underexpansion was high in the Perceval sutureless bioprosthetic valve but overall underexpansion was not associated with HALT. Patients with an overall expansion < 80% had a higher incidence of HALT though not significantly. To determine if severe oversizing increases the risk for HALT in this prosthesis requires further study. Regional underexpansion in the Perceval sutureless bioprosthetic valve was not associated with HALT.
